# Circadian Host-Microbiome Interactions in Immunity

**DOI:** 10.3389/fimmu.2020.01783

**Published:** 2020-08-14

**Authors:** Thomas D. Butler, Julie E. Gibbs

**Affiliations:** Centre for Biological Timing, Faculty of Biology Medicine and Health, University of Manchester, Manchester, United Kingdom

**Keywords:** microbiome, circadian, immunity, diurnal, short chain fatty acids

## Abstract

The gut microbiome plays a critical role in regulating host immunity and can no longer be regarded as a bystander in human health and disease. In recent years, circadian (24 h) oscillations have been identified in the composition of the microbiota, its biophysical localization within the intestinal tract and its metabolic outputs. The gut microbiome and its key metabolic outputs, such as short chain fatty acids and tryptophan metabolites contribute to maintenance of intestinal immunity by promoting barrier function, regulating the host mucosal immune system and maintaining the function of gut-associated immune cell populations. Loss of rhythmic host-microbiome interactions disrupts host immunity and increases risk of inflammation and metabolic complications. Here we review factors that drive circadian variation in the microbiome, including meal timing, dietary composition and host circadian clocks. We also consider how host-microbiome interactions impact the core molecular clock and its rhythmic outputs in addition to the potential impact of this relationship on circadian control of immunity.

## Introduction

The circadian clock is a critical regulator of immunity, including homeostatic processes and responses to immune challenge (1–3). It is now very well established that intrinsic clocks within cells of the immune system regulate their function, influencing the manner in which they respond to a pathogen. However, extrinsic rhythmic signals also drive rhythmic behavior in immune cells. The gut microbiome is recognized as a major influence over the immune system, impacting on its development and daily function. Work over the last decade has established that this bacterial community exhibits 24 h oscillations in composition, biophysical localization and function. Furthermore, these oscillations are critical for driving rhythmic metabolic processes both within the gut and further afield in organs such as the liver. Here we examine the potential impact of circadian rhythms in the microbiome on immunity, and pose the question might rhythms in the microbiome influence circadian control of immunity?

### The Circadian Clock

Entrainment to the 24 h environment is vital to allow organisms to temporally arrange their daily functions (such as feeding, metabolism and sleeping) to align with optimal conditions. Nearly every cell of the body (including those of the immune system) contains the molecular machinery required to keep the clock ticking (4). However, organisms cannot set the time correctly without the help of environmental cues termed “zeitgebers” (meaning time-givers). The most important zeitgeber is the light-dark cycle, which entrains the central clock within the suprachiasmatic nucleus (SCN). Other zeitgebers, such as food availability (5), hormones (6), and body temperature (7) are also important, particularly for influencing peripheral pacemakers (8). Peripheral clocks are synchronized by the SCN, but continue to oscillate when uncoupled from the central clock, highlighting their ability to respond autonomously to changes in the local micro-environment (3, 9, 10).

The molecular clockwork machinery relies on transcriptional-translational feedback loops (TTFL), the duration of which dictates the period of the circadian rhythm. The primary TTFL involves activator proteins circadian locomotor output cycles kaput (CLOCK) and brain muscle arnt-like 1 (BMAL1) interacting with repressor proteins PERIOD and CRYPTOCHROME (4). CLOCK and BMAL1 heterodimerise and bind Enhancer-box (E-box) sequences on target genes for PERIOD and CRYPTOCHROME, which accumulate in the cytoplasm before translocating to the nucleus to inhibit further CLOCK-BMAL1 activity by negative feedback (11). The main TTFL is stabilized by an auxiliary feedback loop involving REV-ERBα and retinoid-related orphan receptor (ROR)α, which repress and activate BMAL1, respectively (12). In this review, we consider bi-directional interactions between the circadian timing system and the gut microbiome and how this influences immune function.

### The Microbiome

The microbiome is defined as an environment containing a heterogeneous community of microbes (including bacteria, fungi, and viruses) and the functional consequences of these microbes, such as their metabolic outputs and interaction with the host. By contrast, microbiota solely describes the community of organisms in isolation (13). It is generally accepted that the fetus develops in a sterile environment and first contact with microbes occurs during birth. An infant's microbiome is influenced by delivery route and method of feeding (14). The first 2–3 years of life oversee significant flux in the composition of the gut microbiome before assuming an adult-like profile, influenced by cessation of breast-feeding, increasing complexity of dietary nutrients and environmental exposures, including antibiotics (15, 16).

The advent of high throughput genome-sequencing technology and large scale projects such as the National Institute for Health's Human Microbiome Project (17) and the European Commission's Metagenomics of the Human Intestinal Tract (18) have dramatically improved our understanding of the phylogenic composition and variability of the microbiota, which in humans has over 150 genera from three main phyla: Firmicutes, Bacteroidetes and Actinobacteria (19). The abundance of these phyla varies significantly between individuals (20, 21).

Our understanding of microbiome function is growing constantly, with main areas of interest in development, immunity and metabolism. Here we focus on the impact of gut microbiota rhythmicity in directing immunity.

### The Gut Microbiome Shapes Immunity

The gut microbiota contribute to the development, maturation and regulation of the host immune system. The importance of the microbiome in shaping the immune system is perhaps best demonstrated by studies with germ free (GF) animals. GF mice have underdeveloped gut-associated lymphoid tissues, fewer and smaller Peyer's patches and mesenteric lymph nodes, and impaired development of isolated lymphoid follicles (22, 23). GF mice have reduced levels of secretory IgA in the intestine (24), and the morphology of the intestinal epithelial cells (IECs), which are normally in direct contact with the microbiota, is modified, with altered microvilli formation and slower cell turnover (25). Furthermore, the absence of a microbiota is associated with arrested capillary network formation within the intestines (26, 27).

Studies with GF mice elegantly demonstrate that commensal bacteria are essential for the development of immune cell subsets, both within the gut lamina propria and further afield. The absence of gut commensals leads to defects in circulating innate immune cell populations (including neutrophils, monocytes and macrophages) and cells within systemic immune sites (including the spleen, bone marrow and liver) as well as delayed neutrophil aging (28–30). Much focus in the field has been surrounding the influence of the microbiota on shaping T cell populations, although it is clear that this influence extends much further to include B cells and innate immune cells. Work over the last decade has assigned roles for individual commensal species in influencing the composition of the lamina propria T lymphocyte subsets. For example, in rodents, segmented filamentous bacteria (SFB) induce intestinal T helper 17 (T_h_17) cells (31, 32), and in humans *Bifidobacterium adolescentis* plays a similar role (33). GF mice have reduced numbers of regulatory T cells (Tregs) in the lamina propria, which can be rescued by re-colonization with strains of *clostridium* (34). These studies highlight the importance of the microbiota for maintenance of the T_h_17/Treg axis. In support, outcomes of mouse models of autoimmunity are often dependent on colonization status. For example, GF mice exhibit marked attenuation in murine models of experimental arthritis (35), experimental autoimmune encephalomyelitis (EAE) (36) and uveitis (37). In the case of EAE (a model of CNS inflammation), the absence of the microbiota is associated with loss of susceptibility as a consequence of perturbations in the balance of Tregs and T_h_17 cells. This balance is restored after re-colonization with SFB and susceptibility returns (36). Similarly the reduction in T_h_17 cells associated with GF mice results in an attenuated arthritis in the K/BxN model of experimental arthritis (35). Intriguingly, in uveitis, retina-specific T cells have been detected in the intestine. Given that the eye is immunologically privileged, it may be that the gut microbiome is playing a role in antigen-mimicry, whereby T cells are triggered by a surrogate antigen present in the gut environment, mimicking a retinal antigen (38). The existence of a gut-retina axis is supported by the presence of uveitis in a subset of patients with inflammatory bowel disease, although further work is required to implicate the microbiome directly (39).

Murine models of autoimmune-driven Type 1 diabetes mellitus, such as non-obese diabetic (NOD) mice are driven in part by activation of innate immune component toll-like receptors (TLR) (40). NOD mice with genetic deletion of *MyD88*, a common adaptor protein for TLR signaling, are protected from type 1 diabetes, but this protection is lost in GF conditions (41). In keeping, NOD mice treated with antibiotics from an early age demonstrate an increased incidence of type 1 diabetes mellitus (42, 43). Together, these data suggest the microbiome modulates innate immunity through TLR signaling to impact risk of autoimmune metabolic diseases such as diabetes in mice.

### Metabolic Outputs of the Microbiota Direct Immunity

**Figure 1:**
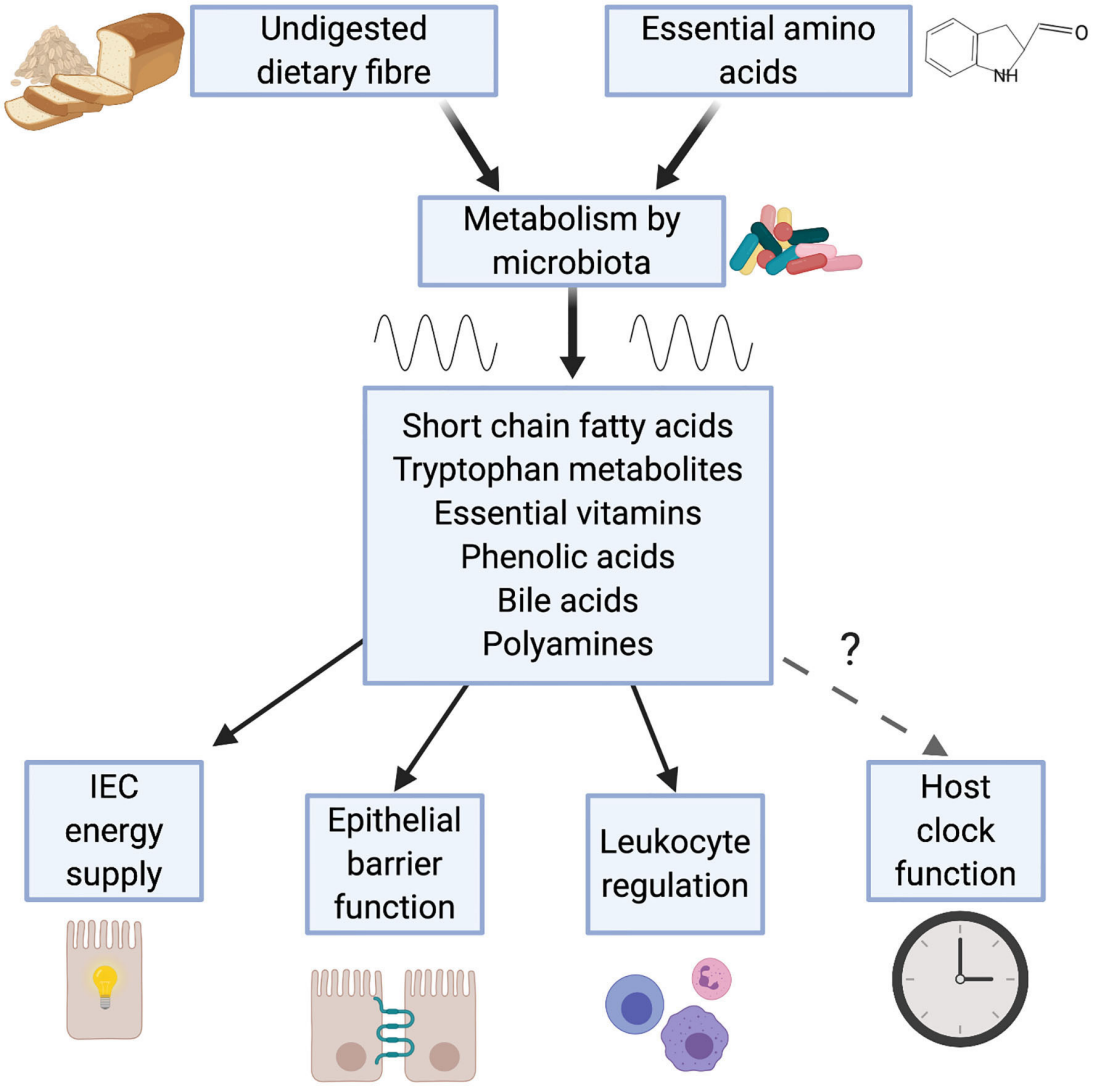
Metabolic outputs from the microbiome influence immunity. Bacteria within the gut breakdown substances derived from the diet. Emerging evidence demonstrates that production of a number of these metabolites is rhythmic, a consequence of feeding rhythms and rhythmic function of the microbiome. Many of these microbial metabolites (e.g. short chain fatty acids, tryptophan metabolites and bile acids) play a key role in immunity, contributing to: intestinal epithelial cell (IEC) function; maintenance of leukocyte populations such as regulatory T cells and macrophages and intestinal tolerance.

Many of the effects the microbiome has on immunity are attributable to the metabolic outputs of the gut microbiota (44, 45). Intestinal bacteria produce a wide range of metabolites, including short chain fatty acids (SCFAs), tryptophan metabolites, essential vitamins, phenolic acids, polyamines and bile acids, which act to modulate host metabolism and immunity (Figure 1).

#### Short Chain Fatty Acids

SCFAs [for example, acetate (C_2_), proprionate (C_3_), and butyrate (C_4_)] are volatile fatty acids composed of a backbone of between 1 and 6 carbon atoms. Derived from bacterial anaerobic fermentation of dietary fibers in the colon, they promote intestinal epithelial barrier function and regulate the host mucosal immune system. SCFAs are important for maintenance of IEC turnover and barrier function (46–48). Butyrate is a critical source of energy for IECs (49), and also regulates expression of the transcription factor *hypoxia-inducible factor* (*HIF)1*α, which acts to co-ordinate barrier function (46) and induces IL10RA, which promotes expression of the tight junction protein Claudin 2 (48). In addition, SCFAs act on multiple gut resident immune cells to facilitate maintenance of intestinal immunity. The earliest observation of the role for SCFAs in maintenance of immune cell populations was made in Tregs. Butyrate and proprionate facilitate extrathymic generation of Tregs (50, 51) by enhancing histone H3 acetylation in the promoter and conserved non-coding sequence regions of the FoxP3 locus (52). More recently it has been established that the effects of SCFA extend far beyond these anti-inflammatory cells to influence the function of both adaptive and innate immune cell populations. These include macrophages (53, 54), B cells (55), CD8^+^T cells (56), type 3 innate lymphoid cells (ILC3s) (57, 58) and neutrophils (58). For example, butyrate alters the metabolic behavior of intestinal macrophages, promoting a state of alternative activation and thus promoting microbial tolerance (53).

#### Tryptophan Metabolites

Tryptophan is a dietary essential amino acid metabolized in the gastrointestinal tract via three different pathways: the kynurenine pathway; the serotonin pathway; or via direct metabolism by microbiota (59). Whilst the former two metabolic pathways are regarded as host-dependent, the microbiota has been shown to influence them (59). Tryptophan metabolism by the microbiota includes the transformation of tryptophan to indole and its derivatives, many of which are ligands for the aryl hydrocarbon receptor (AhR). This includes: indole-3-aldehyde (IAId), indole-3-acetate (I3A), indole 3-proprionic acid (IPA), indole-3-acetaldehyde (IAAId), 5-hydroxyindole-3-acetic acid (5-HIAA) and indoleacrylic acid.

**Figure 2:**
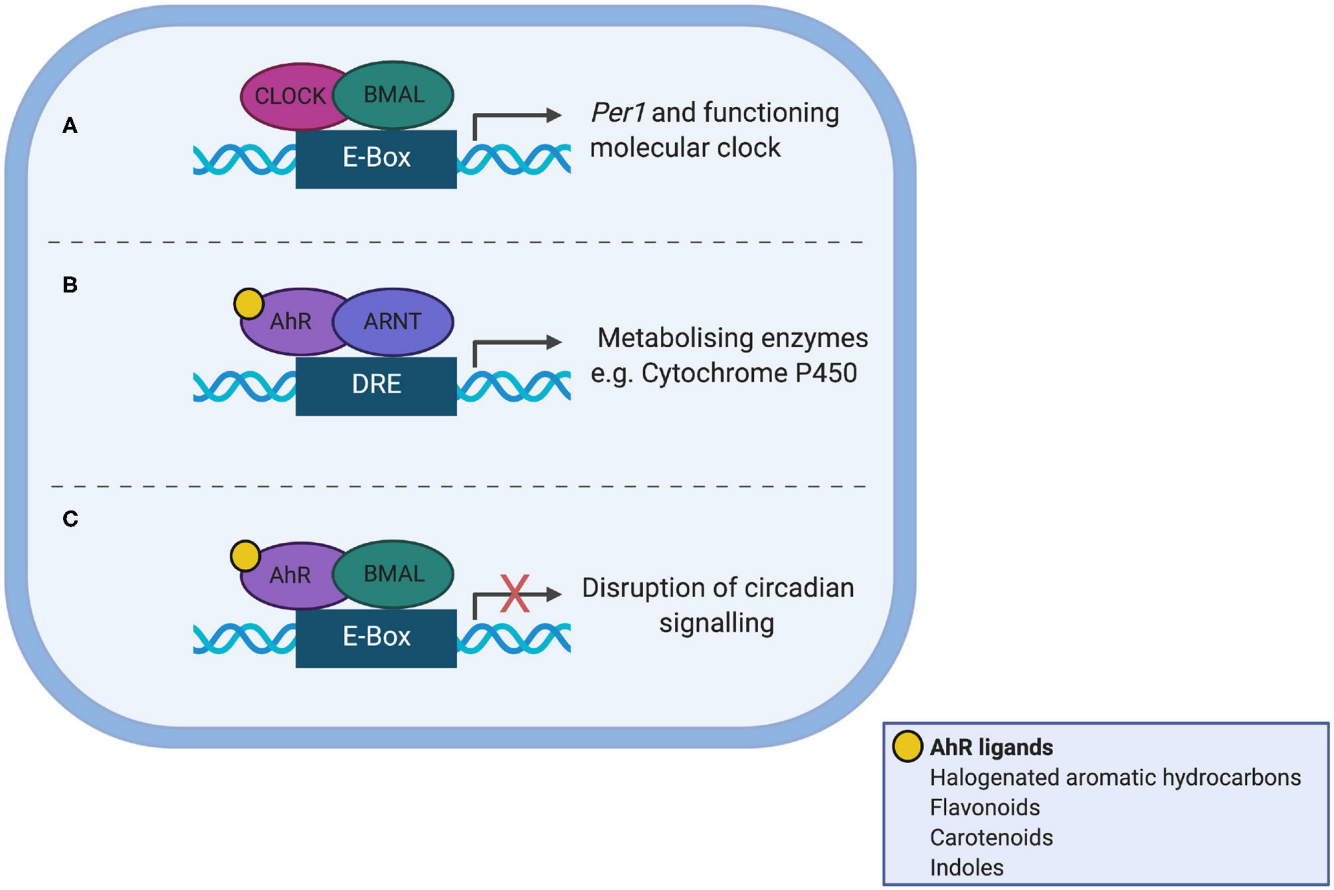
Interaction between the aryl hydrocarbon receptor (AhR) and the molecular clock. **(A)** In normal circadian clock function, CLOCK and BMAL heterodimerise and bind the E-box promotor, to transcribe *Per1*. **(B)** AhR shares sequence homology with CLOCK. Ligand-bound AhR heterodimerises with aryl hydrocarbon receptor nuclear translocator (ARNT) in the nucleus leading to transcription of enzyme superfamilies. **(C)** Ligand-bound AhR can also heterodimerise to BMAL1, which disrupts normal binding of CLOCK/BMAL to the *Per1* promoter, leading to circadian disruption.

AhR is a Per/Arnt/Sim (PAS) domain protein and a fundamental modulator of immunity, controlling the differentiation and inflammatory potential of innate and adaptive immune cells at the gut barrier and systemically (60, 61). AhR shares significant sequence homology to the core clock protein CLOCK and as such offers itself as a nodal point between the gut microbiota, circadian clocks and immunity (Figure 2). Ligand bound AhR heterodimerises with aryl hydrocarbon receptor nuclear translocator (ARNT) in the nucleus, but can also heterodimerise with BMAL (62) to disrupt normal binding of CLOCK/BMAL to the *Per1* promoter (63). AhR plays a role in regulating circadian rhythms in behavior and physiology, as loss of one allele alters responses to changes in the light dark cycle, increases the amplitude of hepatic core clock genes and alters rhythms in glucose and insulin (64). Conversely, AhR activation alters rhythmic expression of core circadian regulators within the periphery; for example chronic administration of the AhR agonist 2,3,7,8-tetrachlorodibenzo-p-dioxin (TCDD) dampens rhythmic expression of core clock genes and clock-controlled hepatic genes disrupting circadian regulation of hepatic metabolism (65). To date there is no direct evidence that microbiota-derived tryptophan metabolites affect timing mechanisms, but this is a certainly a possibility.

Tryptophan metabolites produced by the microbiota are important for regulation of immunity. For example, IAId produced by lactobacilli induces IL22 production to support antifungal resistance and ameliorate colitis in mouse models (66). I3A reduces the production of pro-inflammatory cytokines by macrophages and slows their migration toward a chemotactic signal in an AhR dependent manner (67). Additionally, tryptophan metabolites such as IPA act via pregnane X receptors (PXR) to regulate intestinal barrier function through TLR4 activity (68, 69). Recent work has uncovered a mechanism whereby bacteria derived butyrate enhances production of 5-HIAA which acts via AhR to support regulatory B cell function (70). This highlights the importance of interactions between different microbial species to promote immunity.

#### Bile Acids

Bile acids (BAs) are cholesterol-derived molecules produced in the liver and secreted into the duodenum. Whilst the vast majority (~95%) are re-absorbed through the terminal ileum, several different phyla of gut bacteria can transform the remainder to secondary bile acids, with immune modulatory effects. Recently there has been a flurry of activity in this field ascribing a role for secondary BAs in T cell biology. The lithocholic acid (LCA) derivatives 3-oxoLCA and isoalloLCA regulate T_h_17 and Treg cell differentiation in the lamina propria (71) and RORγ^+^ Tregs in the colon (72). Furthermore, isodeoxycholic acid (isoDCA) promotes the generation of peripheral Tregs (73).

### Evidence for Intrinsic Circadian Clocks Within Gut Bacteria

The existence of intrinsic circadian clocks has rarely been demonstrated in organisms outside of the eukaryotic kingdom, with the exception of certain photosynthetic cyanobacteria. *Synechococcus elongatus* utilizes 3 clock proteins (KaiA, KaiB and KaiC) in a post-translational oscillator cycle of phosphorylation and de-phosphorylation to generate 24 h rhythms (74). The *S. elongatus* clock can synchronize to 24 h temperature cycles and directs clock-regulated gene expression of almost the entire genome (75, 76). It appears that it is not necessary to have all three Kai proteins in order to exhibit rhythmicity. *Rhodobacter sphaeroides* are purple photosynthetic bacteria which express self-sustaining rhythmicity under aerobic condition but lack *KaiA* (77), suggesting that either KaiB and KaiC function differently from those in Cyanobacteria, or that there may be novel components regulating the clock mechanisms in place here.

The existence of timekeeping components in non-photosynthetic bacteria has been addressed in part by exploring whether homologs of the *Kai* genes exist in other bacteria. Whilst *KaiB* and *KaiC* are quite highly conserved, *KaiA* is missing in many other prokaryotes (78). However, like *R. Sphaeroides*, circadian cycles can still be still displayed as in the case of the marine cyanobacterium *Prochlorococcus* (77). *Enterobacter aerogenes* is the first gut commensal bacteria found to express 24 h rhythms outside the host (79). This gram-negative motile bacterium from the phylum Proteobacteria shows circadian rhythmic swarming behavior in the presence of melatonin, which was unreproducible in other gram-negative bacteria such as *Escherichia coli* or *Klebsiella pneumoniae*. Furthermore, transformation with a flagellar motor-protein-driven luciferase reporter reveals temperature-compensated circadian rhythm of luciferase activity synchronized by melatonin (80). Given the abundance of extrapineal melatonin secreted within the gut (81), these studies present this diurnal hormone as a potential zeitgeber for the gut microbiome and may present one mechanism of host control of microbiome rhythmicity. Further studies are required to understand the intrinsic circadian mechanisms of *E. aerogenes* and explore the presence of intrinsic rhythms within other species of the gut microbiota. One possibility is that gut commensals have evolved to respond rhythmically to environmental signals through horizontal gene transfer with human hosts (80, 82). The presence of intrinsic circadian machinery in gut microbiota would have far-ranging implications for understanding host-microbiome interactions.

### Rhythmicity of the Microbiome

With the discovery of resident intestinal clocks (83–85) and rhythmicity in multiple immune cells, interest is growing in the circadian influence on host-microbiome interaction and immunity. 16S rRNA gene sequencing of the fecal microbiome has demonstrated diurnal oscillations in the relative abundance of roughly 15% of OTUs, accounting for around 60% of the murine microbiome, with corresponding oscillation in 10% of OTUs in humans (86, 87). In addition rhythmicity in total microbiota has been described, driven predominantly by Bacteroidetes, which peak during the murine active phase (88). Diurnal oscillations in abundance also exist robustly in healthy humans despite marked inter-human variation in gut microbiome composition (20).

The potential functional consequences of a rhythmic microbiome have been highlighted via metagenomic studies showing oscillations in up to a quarter of total microbiome genetic material in both mice and humans (86). KEGG pathway analysis identified time-of-day grouping of functions such as cell growth and energy metabolism during the murine dark phase, with environmental sensing and flagellar assembly during the light phase (86). Human metagenome analysis demonstrated inversion of peaks in pathway activity compared to nocturnal mice (86). Interestingly, in a cohort of patients with Type 2 diabetes mellitus, diurnal oscillations in microbiome composition were dampened with metagenomic analysis highlighting impact on microbial pathways that process amino acids and fatty acids (89). This is notable as one of the first studies to associate an arrhythmic microbiome with a human disease state.

Diurnal oscillations have been detected in the biophysical distance between mucosal bacteria and IECs, with closest proximity during the murine active, dark phase (90). In mice with global knockout of RegIIIγ, an antimicrobial peptide (AMP) secreted by IECs into the mucus layer, rhythms in the mucosal microbiome were attenuated, suggesting AMPs have a part to play in control of mucosal rhythmicity as well as epithelial cell-microbe distance (90, 91). The mucosal microbiome in closest proximity to the epithelial barrier is most likely to be sampled by mucosal immune components and co-ordinated rhythmicity between the host and microbiome will likely produce the most effective balance in gut homeostasis between tolerance and activated immune response. Thus, whilst the fecal microbiome is easy to sample, the mucosal microbiome may provide more answers to the links in circadian host-microbiome interactions.

Metabolic outputs of the microbiota also exhibit diurnal rhythmicity (Table 1). This is heavily influenced by feeding rhythms of the host providing targets for metabolism, but rhythms in microbial activity clearly also contribute. SCFA levels show diurnal fluctuations in concentration in feces, caecal samples and plasma (92–94). These oscillations are regulated by feeding time as rhythmicity in caecal SCFA are abolished in arrhythmic *Bmal1*^−/−^ mice, but restored by a restricted feeding regimen (94). Oscillations are also sensitive to dietary composition as a high-fat diet (HFD) abolishes rhythms in fecal butyrate (92). While levels of liver-derived conjugated bile acids exhibit post-prandial fluxes during the day in humans, levels of unconjugated bile acids peak late at night, likely reflecting their dependence on microbial activity (95). Many other microbiota-derived or microbiota-modulated metabolites exhibit diurnal rhythms in the intestines and serum, including the polyamine ornithine and the amino acid proline (90, 96). To date, the impact of these rhythms on downstream immunological processes has not been explored, but the importance of microbial metabolites in host immunity makes this an important area to explore.

**Table 1:** Microbial metabolites with demonstrated daily rhythmicity in tissue or circulating concentrations.

**Metabolite**	**Species**	**Tissue**	**References**
**Short chain fatty acids**		
Butyrate	Mouse	Feces	(92)
	Mouse	Feces	(93)
	Mouse	Plasma	(94)
	Mouse	Caecum	(94)
Acetate	Mouse	Feces	(93)
	Mouse	Plasma	(94)
	Mouse	Caecum	(94)
Proprionate	Mouse	Feces	(93)
	Mouse	Plasma	(94)
**Bile acids**		
Unconjugated Bile acids	Human	Serum	(95)
**Amino Acids**		
Threonine	Mouse	Serum	(90)
Ornithine	Mouse	Serum	(90)
Proline	Mouse	Serum	(90)
α-aminobutyric acid	Mouse	Serum	(90)
**Vitamins**		
Biotin	Mouse	Caecum	(90)
Lactate	Mouse	Caecum	(90)
**Polyamines**		
Spermidine	Mouse	Serum	(96)

Timing of food intake is a circadian process centrally controlled by the host and influenced by hunger, food availability and social and cultural norms (97). Mice with disrupted circadian light entrainment show a loss of fecal microbiome diversity, loss of microbiome oscillations, increased weight gain and impaired glucose tolerance despite similar calorie intake and energy expenditure to controls on a standard light:dark cycle (98–100). In a “jet-lag” model, mice phase-shifted by 8 h every 3 days for 4 weeks demonstrate attenuated diurnal oscillations in microbiome abundance, likely driven by loss of diurnal food intake (86).

**Figure 3:**
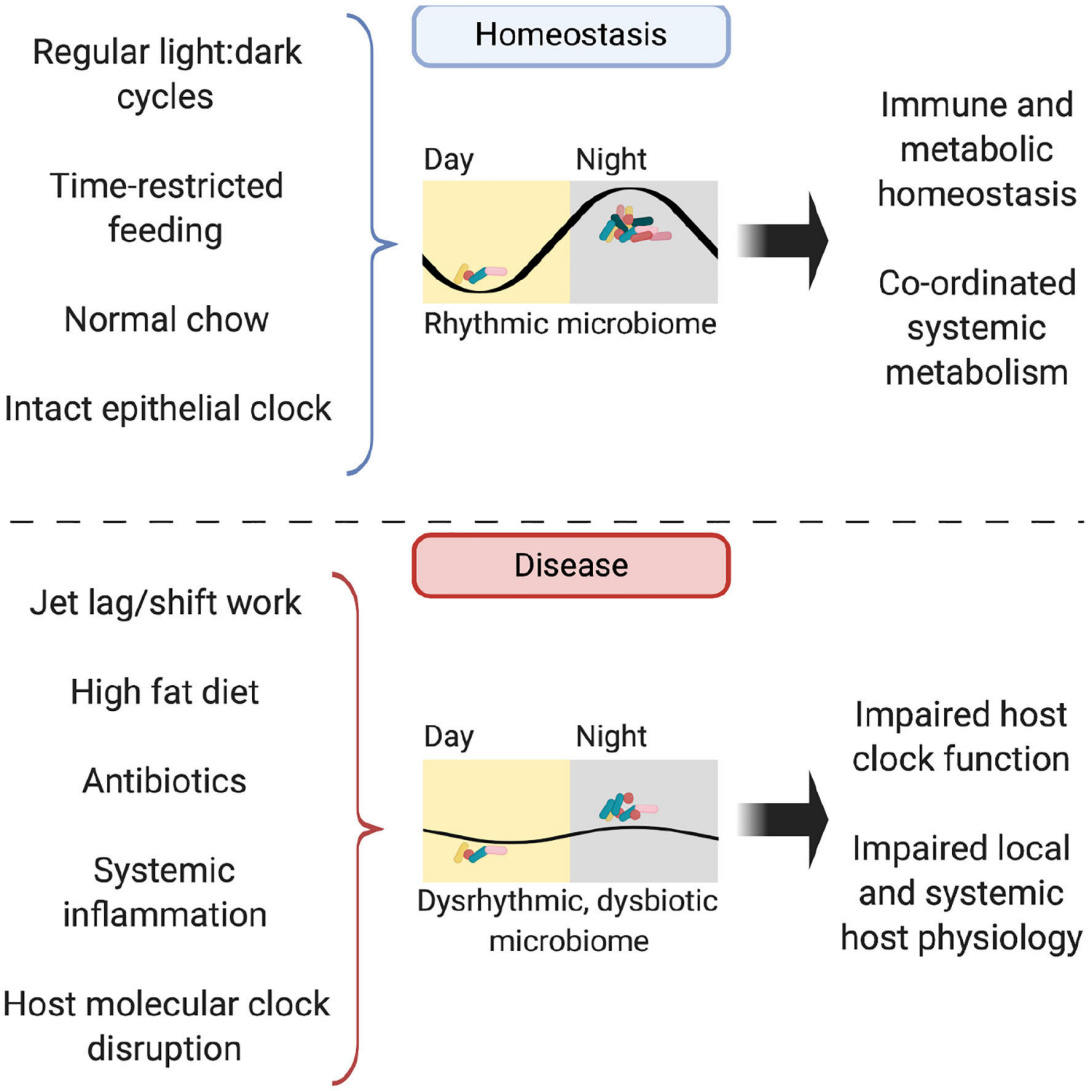
The rhythmic microbiome. In homeostasis, the microbiome is rhythmic in its composition, abundance and biogeographic distance from the intestinal mucosa, with its abundance peaking in the murine dark, active phase. Possible entraining factors include light:dark cycles, timing and composition of food intake and host molecular clock function. Functioning oscillations in the microbiome drive homeostasis in both immunity and metabolism, both locally in the intestine and further afield in organs such as the liver. In disease, disruption to entrainment via jet lag, high fat diet, illness and treatments such as antibiotics lead to perturbations in microbiome rhythmicity and subsequent impairment of local and systemic immune-metabolic homeostasis.

In addition to timing of food, dietary composition also impacts the microbiome rhythmicity. The availability of high-fat diet *ad libitum* drives mice to lose their diurnal eating habits and spread their intake through the day (101). This dampens diurnal oscillations in the microbiome (which are restored when time-restricted feeding is applied) and results in persistent microbial dysbiosis (87). Jet-lagged mice fed a high-fat diet experience greater weight gain compared to non-shifted controls, despite equal food intake; a phenotype attenuated by concurrent antibiotic administration or provision of a high-fat diet to GF mice (86, 92). Fecal microbial transplantation (FMT) from mice with jet lag to GF, non-shifted mice conferred an obesity phenotype not seen in FMT of non-jet-lagged mice (86). This suggests that hosts with a disrupted clock are more susceptible to harboring a dysbiotic microbiome, which is sufficient to drive adverse metabolic consequences for the host (Figure 3).

### The Host Clock Shapes the Microbiome

Whilst feeding times clearly drive rhythms in the microbiome, the host circadian molecular clock also plays a role, with studies evidencing the importance of the biological clock in regulating the composition of the microbial community as well as the rhythmicity. Studies utilizing *Cloc*k^Δ19^ mice (as a genetic approach to model circadian disruption) report increased intestinal hyperpermeability (102) and reduced microbial diversity (103) of the gut microbiome. It is feasible, but as yet undemonstrated, that these changes to the microbiota influence barrier integrity (or *vice versa*). To address the contribution of the host clock in driving microbiome rhythmicity, studies often use transgenic mice lacking a functional clock. Most studies here have been undertaken using animals with global deletion of core clock genes, which complicates interpretation of results as this likely leads to arrhythmic feeding behavior. Mice with global *Bmal1* knockout are arrhythmic and demonstrate attenuated compositional microbiome rhythmicity, whilst retaining circadian oscillations in total fecal microbiome abundance (88). Arrhythmic mice with global *Per1/2* deletion lose rhythmicity in abundance of fecal and mucosal microbiomes as well as metagenomic and metabolic outputs (86, 90). In addition, the intestinal microbiome of global *Per1/2* knockout mice is dysbiotic with less diversity compared to littermate controls (86). Instigation of a time-restricted feeding schedule to *Per1/2* knockout mice to align food consumption with wildtypes recovers oscillations in the microbiome (86). Targeting the molecular clock in specific cells will further delineate the relative contributions of central host rhythms and circadian rhythms in local mucosal immunity to microbiome dysbiosis. Indeed, this approach has revealed a subset of ILCs as critical regulators of the rhythmic microbiome.

#### ILCs as Rhythmic Regulators of the Microbiome

ILCs are circadian rhythmic tissue-resident lymphoid cells. There are currently five subsets of ILCs, which align with their T helper cell counterparts comprised of natural killer cells, ILC1s, ILC2s, ILC3s and lymphoid tissue inducer cells (104). ILC3s are innate counterparts to T_h_17 cells, characterized by expression of RORγt and production of IL17, IL22, and GM-CSF and are the predominant ILC class found at mucosal sites such as the intestine. ILC3s regulate the composition of the microbiota by encouraging preferential commensal growth whilst contributing to removal of pathogens. ILC3-derived IL22 binds directly to IECs and acts through Signal Transducer and Activator of Transcription (STAT)3 to support a multitude of functions that bolster the luminal biophysical barrier including production of AMPs, goblet cell differentiation and subsequent mucus production (105, 106). In addition, IL22 improves colonization resistance by fucosylating IEC-associated proteins that commensal bacterial preferentially use as an energy source, thus helping commensals out-compete pathogens (107, 108).

**Figure 4:**
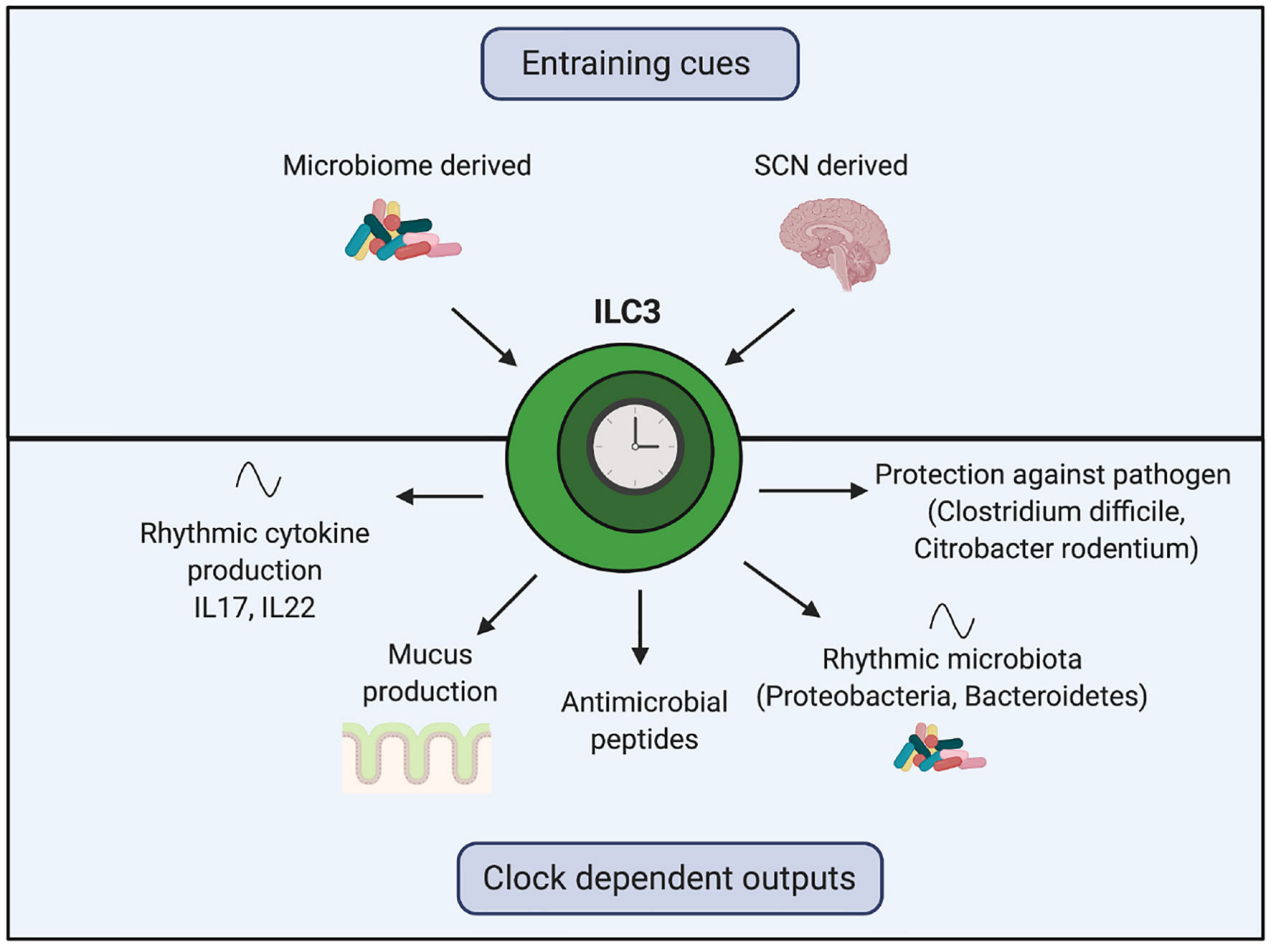
The role of the ILC3 clock in regulating the rhythmic microbiome and barrier function. Clocks within ILC3s are synchronized by the SCN and local microbiome-derived cues. Genetic manipulation of core clock genes within ILC3s demonstrates the importance of this intrinsic clock for regulating crosstalk between the host and the microbiome. Loss of the ILC3 clock results in decreased gene expression of anti-microbial peptides, reduced mucus production, disrupted oscillations in components of the microbiota and cytokines and impaired responses to harmful bacteria.

Recent work has uncovered the importance of the circadian clock on the function of ILC3s (Figure 4). In homeostasis, ILC3s exhibit oscillating expression of core clock genes, transcription factors (such as *Ahr)* and functional genes (such as *il17* and *il22)* (109–111). ILC3-specific knockout of either *Bmal1* or *Rev-erb*α decreases intestinal ILC3 numbers, decreases gene expression of anti-microbial peptides and mucus production and disrupts oscillations of Proteobacteria and Bacteroidetes in the microbiome (109–111). Intriguingly, these genetic manipulations do not affect abundance of ILC3s in the spleen and lung, suggesting intestinal ILC3s may be uniquely reliant on *Bmal1* (109).

Mice with *Rev-erb*α-deficient ILC3s exposed to *Clostridium difficile* show an exaggerated IL17 response and a higher bacterial burden (111). Overproduction of IL17 and IL22 in *Bmal1*-deficient ILC3s is abrogated by antibiotic treatment (110). Lymphocyte-deficient mice with *Bmal1-*deficient ILC3s are more susceptible to gut inflammation induced by *Citrobacter rodentium*, with increased epithelial barrier permeability and reduced survival (109). In wildtype mice, oral antibiotic treatment shifts the timing of *Per1* expression in ILC3s and dampens IL22 oscillations (109, 110). Together, these observations illustrate the importance of the ILC3-intrinsic circadian clock in regulation of crosstalk between host ILC3s and the microbiome. In humans with inflammatory bowel disease, there is a reduction in ILC3s within inflamed intestine compared to intra-individual non-inflamed tissue, with associated disruption of ILC3 circadian gene expression (110). Of interest, *Rev-erb*α gene expression was elevated in human ILC3s from inflamed tissue (110, 112). These perturbations to the ILC3 clockwork may have downstream effects on management of microbial rhythmicity.

Multiple components of mucosal immunity exhibit rhythmicity, including the microbiome, IECs, dendritic cells and ILC3s, which will likely need to be co-ordinated and entrained for maximal gut homeostasis. It will be pertinent to delineate the local power balance and determine which circadian clocks, local or central, drive rhythmicity in the gut microbiome during homeostasis and the consequences of disease (90, 113–115).

### Rhythms in the Gut Microbiome Affect Rhythmic Processes Elsewhere

Communication between the host and microbiome is bi-directional. Whilst host clocks and rhythmic feeding drive daily variation in the composition of the microbiome, signals derived from the microbiome can influence circadian host physiology. We are now beginning to establish an understanding of how the microbiota and its metabolic outputs effects rhythmic processes.

A significant proportion of rhythmic transcripts within the intestines are influenced by the intestinal microbiota. The dependence of rhythmic expression of the core clock genes on the microbiome appears to be tissue dependent, with depletion of the microbiome affecting clock genes such as *Ror*α and *Rev-*erbα differently in the colon compared to the small intestines. Mukherji et al. mapped the rhythmic transcriptome in ileal IECs, which includes *nuclear factor interleukin 3 regulated* (*Nfil3), tlr* genes and clock genes *Ror*α and *Rev-erb*α*. D*epletion of the microbiome via administration of antibiotics results in the cessation of rhythmic gene expression and a fall in *Tlr* expression, whilst *peroxisome proliferator activated receptor* (*Ppar)*α becomes constitutively expressed, suppressing downstream circadian outputs including *Nfil3*, with subsequent impairment of host metabolism (113). Administration of LPS (as a surrogate for microbiome activation of toll-like receptors) reinstated RORα-driven circadian *Tlr* expression and recovered homeostatic IEC outputs including hypothalamic-pituitary-adrenal axis-independent corticosterone rhythmicity (113). NFIL3 is important in the development and regulation of immune cells including ILCs, macrophages and dendritic cells and may be one pathway for an arrhythmic microbiome to impact host immunity (116–118). In addition, lower NFIL3 levels have been detected in patients with active inflammatory pathologies such as colitis and arthritis, conditions that have been associated with microbiome dysbiosis (117, 119).

Transcriptomic examination of colonic IECs by Thaiss et al. demonstrated that depletion of the microbiota causes loss of rhythms in many pathways (including nucleotide metabolism and cell-cycle pathways) without affecting the core circadian clock genes (90). Intriguingly, this study also noted emerging *de novo* circadian rhythms in genes associated with metabolic pathways, suggesting the host may acquire compensatory oscillatory programmes in the absence of the microbiota. In keeping, depletion of the microbiome resulted in distinct changes to the temporal organization of the chromatin landscape within IECs. The regulation of transcriptional oscillations is dependent on rhythmic bacterial adherence, as mice lacking RegIIIγ (which have an abundant but non-rhythmic mucosal microbiome) showed an overlapping oscillating transcript with antibiotic-treated mice (90).

Further support for a role for the intestinal microbiota in programming daily rhythms in metabolic networks within the gut comes from a study, which found the gut microbiota drives rhythmic recruitment of HDAC3 to chromatin in IECs of the small intestine (120). Resultant HDAC3-NCoR complexes produce synchronized diurnal oscillations in histone acetylation, expression of metabolic genes, nutrient uptake and intestinal lipid absorption. *Bacteroides thetaiotaomicron* is critical here, as mono-colonization of GF mice with this Bacteroide is sufficient to restore rhythms in HDAC3 expression. Interestingly, the mechanism appears to be restricted to the small intestine; within the colon, genome wide acetylation rhythms persist in the absence of the microbiota (90, 120).

**Figure 5:**
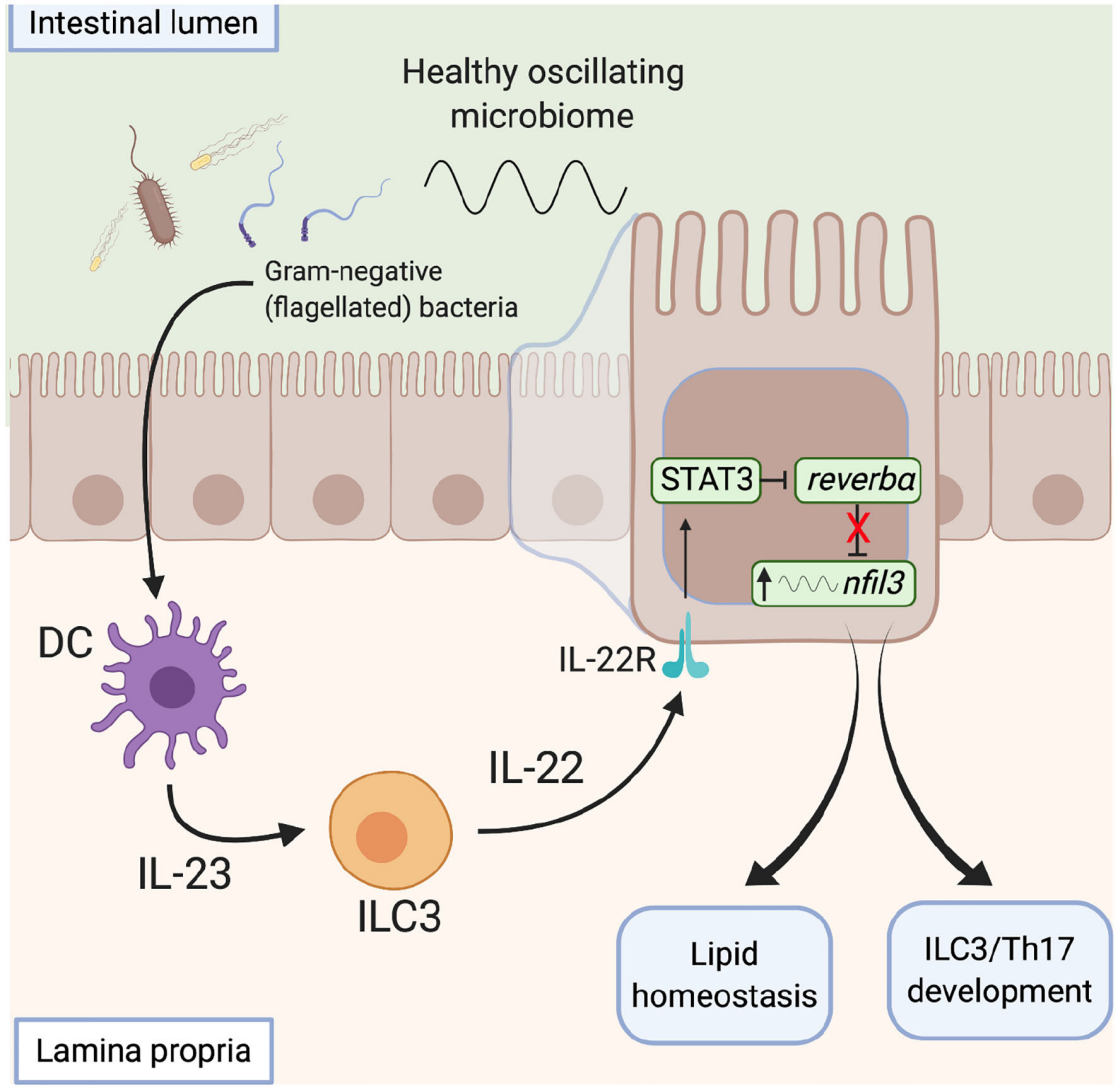
The microbiota influences metabolic programmes within the gut. Signals derived from gram-negative flagellated bacteria resident within a healthy microbiome activate toll-like receptors on lamina propria CD11c^+^ dendritic cells and induce IL23 secretion. In response to increased IL23 levels, ILC3s signal to intestinal epithelial cells via IL22, which activates a STAT3 signaling pathway to repress *rev-erb*α expression, with subsequent de-repression of *nfil3* transcription. Increased *nfil3* expression drives circadian lipid homeostasis and development of ILCs and T_h_17 cells, promoting intestinal homeostasis. DC, dendritic cell. ILC, innate lymphoid cell, STAT, signal transducer and activator of transcription.

The microbiota also influences metabolic programmes within the gut via direct action on the clock. Wang et al. describe a signaling network by which the microbiota modulates clock activity with IECs to influence their metabolic programme (114) (Figure 5). This network integrates local immune cells which respond directly to the microbiota and signal to the IECs. Gram-negative bacteria are detected via toll-like receptors on myeloid cells within the intestinal lamina propria, via a number of potential routes (121). This prompts IL23 release which drives IL22 production by ILC3s. IECs respond to IL22 by up-regulating expression of STAT3, which binds to the promoter of *Rev-erb*α to repress expression. This reduction in REV-ERB levels allows enhanced transcription of *Nfil3*, a transcription factor that regulates a circadian transcription program of genes involved in lipid uptake, immunity and metabolism. In this way, the gut microbiota functions as an entraining cue for systemic processes and thus daily oscillations in the abundance of key bacterial species and/or the biogeography within the gut may be critical here. These studies highlight the ability of the local immune network to sense daily changes in the microbiota and raise the possibility that local immune networks may also be temporally responsive to the microbiota.

It is evident from these studies that the microbiota itself can modulate the core clockwork machinery. It is also now becoming clear that metabolic outputs from the microbiota can directly alter the phase and amplitude of the circadian clock. Oral administration of SCFAs can induce phase changes in peripheral clocks (122). Additionally, metabolites produced by *Clostridium sprogenes* such as [3-(4-hydroxyphenyl) propionic acid (4-OH-PPA) and 3-phenylpropionic acid (PPA)] increase the amplitude and lengthen the period of PER2 oscillations in organotypic SCN slices and other tissues (123). Polyamines (putrescine, spermidine and spermine) can regulate the circadian period of cultured cells and alter the circadian period of mice, likely as a consequence of their ability to promote the interaction between PER2 and CRY1 (96). Finally, secondary bile acids (deoxycholic acid and chenodeoxycholic acid) alter clock gene expression in the ileum, colon and liver (124). Given that some secondary bile acids have been shown to be able to directly bind to RORγt (71), this represents a potential mechanism by which bile acids may modulate the core clock. Together, these findings highlight the possibility that microbiome-derived metabolites act as zeitgebers, perhaps fine-tuning the clock to feeding rhythms.

In addition to the microbiota driving rhythms within the gut, bacterial-derived signals also act further afield to programme circadian networks in the liver (90, 125). The gut microbiome has been identified as a potential driver of HFD-induced changes in liver circadian metabolism (125). The nutritional challenge afforded by HFD drives transcriptional and metabolic reprogramming within the liver, resulting in a gain of rhythms in previously non-circadian transcription factors such as peroxisome proliferative activated receptor γ (PPARγ) (126, 127). Murakami et al. demonstrated that fecal transfer from HFD-fed mice to recipient normal chow fed mice is sufficient to elicit the emergence of circadian rhythms in PPARγ leading to specific rewiring of circadian transcription. Thaiss et al. demonstrated in mice fed normal chow, depletion of the microbiome re-programmes the liver transcriptome, with both loss and gain of rhythmic pathways (90). They went on to identify polyamines and amino acids as contributing microbiome-regulated rhythmic metabolites that signal from the gut to the liver to regulate transcriptional programmes (90). These studies provide proof-of concept that systemic metabolic rhythms are reliant on the rhythmic microbial outputs. It will be interesting to explore the extent to which these rhythmic signals direct other physiological processes, with immune homeostasis being an obvious candidate.

## Conclusions and Future Direction

The intestinal microbiome is very well established as an influential component of immune-metabolic homeostasis. A healthy microbiome exhibits rhythmicity in microbial composition, as well as rhythmicity in its biophysical location and metabolic outputs, each of which will influence immune homeostasis. Whilst evidence is emerging of gut commensals with potential intrinsic rhythmic capacity, it is likely that these microbiome rhythms will be heavily reliant on entrainment derived largely from the host. This may take the form of food timing, food composition, secreted host signals or non-dietary intake including antibiotics and immunomodulatory medication, as well as host disease state. Initial observations of microbiome rhythmicity in immune homeostasis and associated dysrhythmia in inflammatory disease states should drive scientific investigation into the mechanisms of host circadian regulation of the microbiome and *vice versa*, as it is not yet clear which immune components are directly sensitive to changes in microbiome rhythmicity and which host-microbiome pathways predominate in health and disease. Whilst current studies have predominantly focused on the metabolic implications of an arrhythmic microbiome, future work should expand on early work exploring the impact of microbiome rhythmicity on composition and function of immune subsets. Piecing together the relative contribution of each moving part in the microbiome, intestinal epithelium and circulating immune cells will help to understand the role of circadian rhythmicity in crosstalk between the microbiome and immune system.

Evidence of microbiome dysbiosis associated with chronic human inflammatory conditions is growing, however, addition of a circadian component to human immuno-microbiome trials is in its infancy. In humans, manipulation of the microbiome via fecal microbial transplantation is already established to modulate immune response in infective conditions such as *Clostridium difficile* infection and is under investigation in inflammatory conditions such as inflammatory bowel disease (128, 129). It is exciting to consider circadian influence on microbiome health as a pathway to further understanding of mucosal immunology and ultimately improve efficacy of microbiome modulation as a chronotherapy for human inflammatory and auto-immune disease.

## Author's Note

Figures were created with Biorender.com.

## Author Contributions

TB and JG wrote the manuscript and designed the figures. All authors contributed to the article and approved the submitted version.

## Conflict of Interest

The authors declare that the research was conducted in the absence of any commercial or financial relationships that could be construed as a potential conflict of interest. The handling Editor declared a past co-authorship with one of the authors JG.

## Abbreviations

5-HIAA: 5-hydroxyindole-3-acetic acid
AHR: Aryl hydrocarbon receptor
AMP: Antimicrobial peptide
ARNT: Aryl hydrocarbon receptor nuclear translocator
BA: Bile acids
BMAL1: Brain muscle arnt-like 1
CLOCK: Circadian locomotor output cycles kaput
CNS: Central nervous system
EAE: Experimental autoimmune encephalomyelitis
FFAR: Free fatty acid receptor
FMT: Fecal microbial transfer
GF: Germ free
GM-CSF: Granulocyte macrophage colony stimulating factor
GPCR: G-protein coupled receptor
HCAR: hydroxycarboxylic acid receptor
HDAC: Histone deacetylase
HFD: high-fat diet
HIF: Hypoxia-inducible factor
I3A: Indole-3-acetate
IAId: Indole-3-aldehyde
IEC: Intestinal epithelial cell
IL: Interleukin
ILC: Innate lymphoid cell
IPA: Indole 3-proprionic acid
isoDCA: isodeoxycholic acid
LCA: lithocholic acid
LPS: lipopolysaccharide
NFIL3: nuclear factor interleukin 3 regulated
NOD: non-obese diabetic
OTU: Operational taxonomic unit
PAS: Per/Arnt/Sim
PPAR: peroxisome proliferator activated receptor
PXR: Pregnane X receptor
ROR: Retinoid related orphan receptor
SCFA: Short chain fatty acid
SCN: Suprachiasmatic nucleus
SFB: Segmented filamentous bacteria
STAT: Signal transducer and activator of transcription
TCCD: 2,3,7,8-tetrachlorodibenzo-p-dioxin
T_h_17: T helper 17
Treg: Regulatory T cell
TLR: toll-like receptor
TTFL: Transcriptional-translational feedback loop.
